# Whole-exome sequencing analysis in a case of primary congenital glaucoma due to the partial uniparental isodisomy

**DOI:** 10.5808/gi.21044

**Published:** 2022-09-06

**Authors:** Parisima Ghaffarian Zavarzadeh, Morteza Bonyadi, Zahra Abedi

**Affiliations:** 1 Department of Animal Sciences, University of Tabriz, Tabriz 5166-15731, Iran; 2 Laboratory of Systems Biology and Bioinformatics (LBB), Institute of Biochemistry and Biophysics, University of Tehran, Tehran 14155-6619, Iran

**Keywords:** *CYP1B1*, glaucoma, primary congenital, sequence analysis, whole-exome sequencing

## Abstract

We described a clinical, laboratory, and genetic presentation of a pathogenic variant of the *CYP1B1* gene through a report of a case of primary congenital glaucoma and a trio analysis of this candidate variant in the family with the Sanger sequencing method and eventually completed our study with the secondary/incidental findings. This study reports a rare case of primary congenital glaucoma, an 8-year-old female child with a negative family history of glaucoma and uncontrolled intraocular pressure. This case’s whole-exome sequencing data analysis presents a homozygous pathogenic single nucleotide variant in the *CYP1B1* gene (NM_000104:exon3:c.G1103A:p.R368H). At the same time, this pathogenic variant was obtained as a heterozygous state in her unaffected father but not her mother. The diagnosis was made based on molecular findings of whole-exome sequencing data analysis. Therefore, the clinical reports and bioinformatics findings supported the relation between the candidate pathogenic variant and the disease. However, it should not be forgotten that primary congenital glaucoma is not peculiar to the *CYP1B1* gene. Since the chance of developing autosomal recessive disorders with low allele frequency and unrelated parents is extraordinary in offspring. However, further data analysis of whole-exome sequencing and Sanger sequencing method were applied to obtain the type of mutation and how it was carried to the offspring.

## Introduction

Primary congenital glaucoma (PCG) is a severe form of glaucoma characterized by an anatomical defect in the trabecular meshwork in neonates and infants, generally before the age of 3 years [1]. The symptoms are in two primary forms, including milder cases, blurred and partial vision loss in peripheral visibility. Furthermore, it more severely causes secondary symptoms like eyes concretion, night vision loss, and eventually blindness.

Genetic mapping of families with PCG identified the *CYP1B1* (cytochrome P450 family 1 subfamily B member 1) gene locus associated with the *GLC3A* type of disease [2]. Also, the spectrum of mutations in *CYP1B1* varies widely in different populations, depending on geographical context and haplotype. Phenotypical data on 104 unrelated Iranian PCG patients who had previously been screened for *CYP1B1* mutations were analyzed. Patients with *CYP1B1* mutations include 37 male (66.1%) and 29 female (43.9%) (p = 0.30) [2]. It is noticeable to note that the chi-square test or using chi-square contingency tables were used to obtain p-values. These findings show that the overall incidence of PCG in Iran seems to be higher among male subjects and is consistent with data on PCG patients from other populations [2]. The overall PCG occurrence between male patients without harboring *CYP1B1* mutations indicates that other genes or factors can be involved in the PCG phenotypes appearing in a sex-dependent matter [2].

However, PCG occurs up to 10 times more frequently in certain ethnic and geographical groups where consanguineous relationships are common [3]. Hence, these findings with the ethnic differences and the geographical distribution of PCG give us a large *CYP1B1* mutation pattern [3]. An 8-year-old girl was referred to our glaucoma service for uncontrolled intraocular pressure (IOP). The case shows similar symptoms of glaucoma at the early onset without any other set of medical signs and symptoms; evident that the unknown disease might be PCG in the form of non-syndromic. While autosomal recessive rare diseases like glaucoma mainly developed due to the consanguineous relationships in small-scale societies [4]. The parents of this case are unrelated; supposing that she developed glaucoma disease, this was an elusive situation that needed further genetic studies to find the specific genetic reason.

In this study, we aimed to use a variety of genetic testing and experiments investigating specific genetic factors related to eye disorders and manifested phenotype of the case from reliable sources. Hence further studies are performed to detect the type of mutations as hereditary or de novo and, in the case of hereditary, how they are passed down to an offspring from parents [5].

## Methods

### Case presentation

The case was an 8-year-old female patient born in Tabriz-Iran (our investigation was conducted according to the principles expressed in the declaration of Helsinki. In addition, the informed consent form is available at the laboratory of Doctor Bonyadi-University of Tabriz). Whose parents (natural parents) have unrelated marriages and have no phenotype related to eyes disorders or genetic background for PCG. She presented with uncontrolled IOP but no specific medical signs and symptoms. IOP was 30 mmHg and 14 mmHg in the right and left eyes, respectively (measured by a Tono-pen) [6]. In the first stage, the blood sample was obtained for DNA extraction and further analysis. Although there are numerous protocols for nucleic acid extraction, we selected the option which initially involves cell disruption and digestion (with sodium dodecyl sulfate–proteinase K), followed by the addition of salts (6 M sodium chloride with high concentration) [7]. Then the mixture was centrifuged, and the supernatant (containing DNA) was transferred to a new vial and precipitated by applying ethanol [7]. Next, the BGI company (Shenzhen, China) performed whole-exome sequencing (WES) using the patient’s DNA from venous blood.

The overall QC of the raw sequencing data is performed by FastQC [8]. This program provides summaries of sequenced GC content, repetitive sequences, and many other potential anomalies, allowing users to evaluate whether the data have any quality issues [9]. After the conversion of the sequence alignment map (SAM) file to the binary alignment map (BAM) file, the variant calling took place. We received a VCF file including the profile of all variants achieved from the blood sample with WES filtering out low-frequency variations. We only kept the variants that occurred in hotspot regions (such as exonic, splicing, and exonic-splicing sites) and with a frequency of less than 0.05 in significant population studies, including genome-wide association studies (GWAS), 1000 Genome, Genome AD, etc. [10,11]. Later, the remaining variants were investigated through various bioinformatics platforms to observe whether they are pathogenic and related to the phenotype.

After filtering out unspecific variations and studying the rest of them through literature and documents in Clinvar, Intervar, and Varsome platforms [12,13]. Additionally, the secondary structure of mRNA was studied by RTH, the server of RNA SNP [13]. Later, the protein changes were studied by SNP & GO and Provean [14,15]. Ultimately, the enzymatic activity was predicted by Mutpred2 [16]. All the bioinformatics approaches were applied through online servers with reliable p-value explicitly calculated by the server to study the impact of suspicious variations and find the related variant to the disease (written informed consent was obtained from all participants).

As we noticed the majority of genetics research attended by more severe and fatal diseases over recent decades, we ought to consider that a rare disease, namely, glaucoma is the second leading cause of blindness worldwide. Accordingly, it is essential to observe similar symptoms in ocular patients that may develop a subtype of glaucoma and consider further studies on the genetics of the disease to find out more about its characteristics and preventers. Therefore, we presented the first case in Iran with PCG in this study owing to the cytogenetic phenomenon covering uniparental isodisomy.

## Results

The BGI company performed WES using the patient’s DNA obtained from venous blood. Two single nucleotide variants (SNVs) (*CYP1B1*:NM_000104:exon3:c.G1103A:p.R368H) in homozygous state and (NTF4:NM_006179:exon2:c.C263T:p.A88V) in heterozygous state were filtered from WES data. Clinical reports on Clinvar and computational analyzing servers like Intervar and Varsome websites were studied for both variants of *NTF4* and *CYP1B1* genes (Table 1). Further studies declared that the *CYP1B1* variant, which is reported as the variant’s conflicting interpretation of the pathogenicity, is the candidate pathogenic mutation for glaucoma 3A (*GLC3A*) in our case study. Additionally, the effect of the candidate variant on the *CYP1B1* gene was examined under online bioinformatics and computational servers. We report this variant as a pathogenic mutation for GLC3A in this case.

RTH (Center of Non-coding RNA in Technology and Health), the server of RNA SNP applied to identify changes in RNA secondary structure due to the mutation. The p-value defines the range of changes and risks for diseases; our data showed a p-value < 0.02. This proportion means there is no important change in RNA secondary structure (the server obtained the p-value, and here the p-value < 0.02 declare no significant structural change in mRNA).

Since the candidate variant also affected the amino acid sequence (Arg>His), studying protein structures is essential. In this case, we applied SWISS-MODEL online server to compare both 3D structures of the natural and mutated protein. In addition, the effect of amino acid change on protein activity estimated by SNP & GO and Provean servers proved a deleterious and potent relation between the mutation and glaucoma [5,17].

According to the significant functional product of *CYP* family genes as P450 enzymes, we also applied further online analytic studies on the structure of enzymes. MutPred2 provides a comprehensive study on more than 50 features of a specific enzyme. This bioinformatics tool indicated details about the mutation and its effect on disabling the enzyme's allosteric site. Due to the inefficient allosteric site, the enzyme's operation would be deactivated. The score of this mutation is 0.560, and it is “disease-causing” in MutPred2 (p = 0.02) (the server calculated the p-value).

Ultimately it revealed *CYP1B1* (NM_000104) homozygous variant c.G1103A (p.R368H). The child was diagnosed with autosomal recessive glaucoma 3A because of the pathogenic variant (c.G1103A) in the *CYP1B1* gene. The *CYP1B1* variant c.G1103A (p.R368H) creates a nonsynonymous change and is classified as a pathogenic variant according to the American College of Medical Genetics and Genomics (ACMG) guidelines [18].

**Fig. 1.**
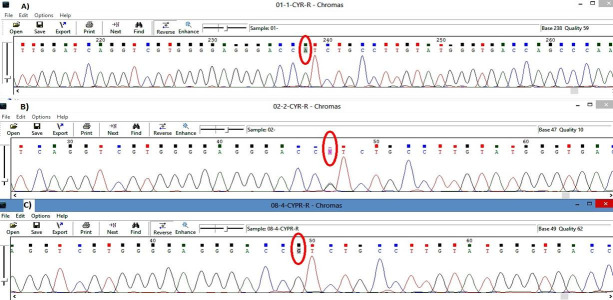
Trio data analysis of the sanger sequencing method in the family. (A) Proband: homozygous state of variation (A/A). (B) Father: heterozygous state of variation G1103>A (G/A). (C) Mother: homozygous state of wildtype (A/A).

Sanger sequence analysis method in the family revealed the pathogenic variant in a heterozygous state in the unaffected father alone (Fig. 1). Evidence shows that *GLC3A* occurred due to the uniparental isodisomy (UPiD) of chromosome 2 [19,20].

In the long run, we completed our study of this case with the secondary findings from WES data. The secondary findings were restricted to the 59 recommended genes for ACMG [21]. All the variations obtained in these 59 genes were thoroughly investigated through the documents, and reports in Clinvar and the selected ones (Table 2) were submitted as pathogenic/likely pathogenic variations in the Clinvar database [22]. Accordingly, *RET* gene (from 59 recommended genes) solely observed variation. Additionally, we studied other candidate variations of our case, which are not placed in recommended genes by ACMG guidelines. However, they show significant risks of being a career for diseases such as familial Mediterranean fever (FMF), a remarkably high prevalence in the region of study (northwest of Iran) [23]. All the studied variants were obtained by extended studying of reports in Clinvar. The discussion section comprehensively explains our bioinformatics analysis and the possibility of their related disorders (Table 2).

## Discussion

*CYP1B1* is a protein-coding gene, and diseases associated with *CYP1B1* include glaucoma 3A, primary congenital, and anterior segment dysgenesis 6. Among its related pathways are arachidonic acid metabolism and drug metabolism - cytochrome P450 [24].

In this case study, the clinical features of a patient with a variant of the *CYP1B1* gene were identified. It is well known that the PCG incidence is higher in populations with high rates of consanguineous marriage, whereas its incidence in western countries is estimated at 1:10,000 [25]. This rate in various inbred populations for which data is available, such as India and Saudi Arabia, ranges from 1:1,200-1:3,300 [25].

By data analysis of the Sanger method in the family members (natural parents and the case), the candidate variant on *CYP1B1* gene c.G1103A (p.R368H) was observed in a heterozygous state in the unaffected father alone. Our evidence illustrated that in this case, developing autosomal recessive glaucoma occurred due to the UPiD of chromosome 2. The computational analysis declares the possibility of transmitting two copies of this variant from the father who is harboring it, which in the genetics area is a similar case of occurring uniparental isodisomy. Therefore by comparing chromosome 2 in parent and offspring, results showed that UPiD occurred between rs4670800 (NM_144713:exon 2:c.G776A:p.G259D) and rs79204362 (NM_000104:exon3:c.G1103A:p.R368H) SNVs on *RMDN2* and *CYP1B1* genes respectively. Other adjacent variants near these positions were heterozygotes, an inevitable situation in the UPiD phenomenon.

**Fig. 2.**
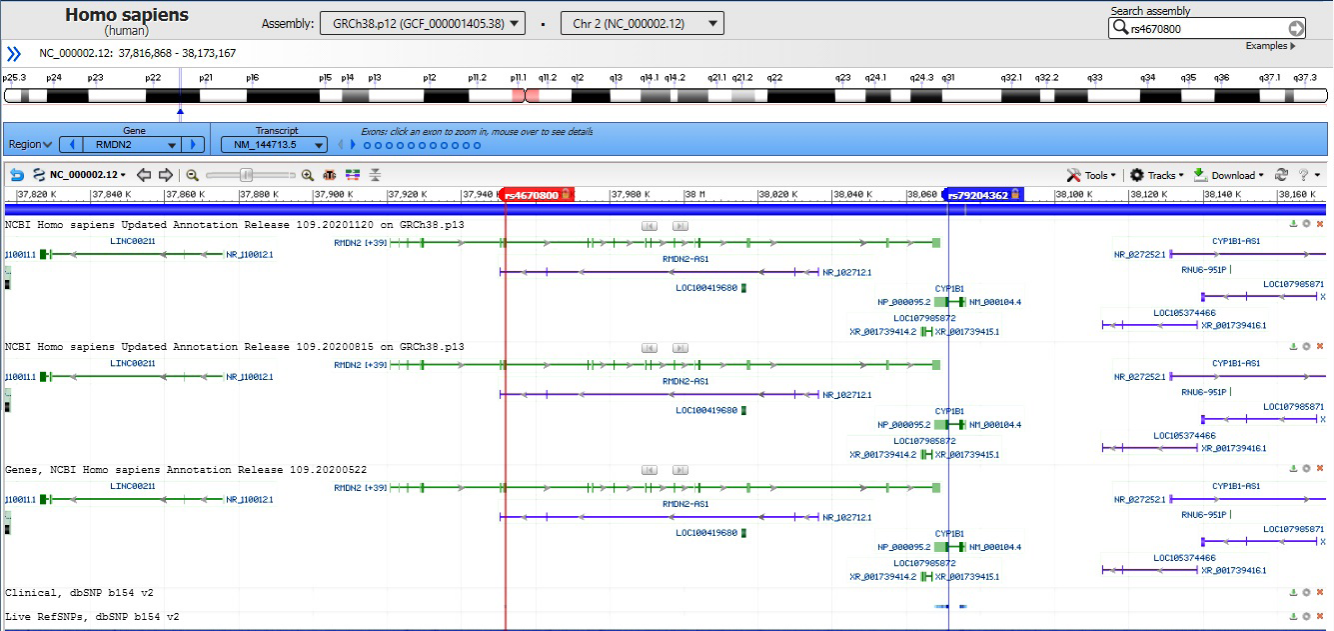
Probable range of uniparental isodisomy in the case observed by Integrative Genomics Viewer (IGV) and the Sanger sequencing method. At intervals of *RMDN2*: NM_144713:exon2:c.G776A:p.G259D (rs4670800) and *CYP1B1*: NM_000104:exon3:c.G1103A:p.R368H (rs79204362).

Our research obtaining the precise location of UPiD was restricted to observations with the Integrative Genomics Viewer (IGV) tool and the Sanger sequencing of the *CYP1B1* gene. The IGV illustrated details that partial UPiD might occur between the two mentioned variants at 2p.22 (Fig. 2) [26].

Additionally, we applied ACMG recommendations of 59 genes for reporting incidental findings [21]. An SNV on *RET* gene was observed as a risk factor for Hirschprung disease. Further investigations obtained other variants that declare the case possible to be a career for *ABCA4*-related disorders: Stargardt disease, Cone-rod dystrophy 3, and retinal dystrophy. And also, for Xeroderma pigmentosum, deficiency of 2-methylbutyryl COA dehydrogenase and phenylketonuria. A pathogenic variant of the *MEFV* gene was observed, which must cause FMF with an autosomal dominant inheritance pattern. Although northwest Iran has a higher abundance of FMF, still in the presence of the pathogenic variant, phenotype has not been observed in the patient. The entire reported variants were submitted multiple times in Clinvar as pathogenic/likely pathogenic variations related to the mentioned diseases and examined with online bioinformatics servers. Ultimately the possibility and risk for the case to be a career are estimated based on the computational findings, though the *RET* variation is a risk factor variant. The variation in the *MEFV* gene causes a prevalent disease in northwest Iran (FMF) with an autosomal dominant inheritance pattern. However, further clues and investigation are necessary to make precise conclusions for the incidental/secondary findings (Table 2).

The diagnosis was made based on molecular findings of WES data analysis. Therefore, the clinical reports and bioinformatics findings supported the relation between the candidate pathogenic variant and the disease. However, it should not be forgotten that PCG is not peculiar to the *CYP1B1* gene.

Although the attention to diagnosing PCG based on the clinical findings is significant, the genetic tests and bioinformatics procedures could also provide priceless information. Several efforts have been initiated to understand the underlying mechanisms of the disease by using GWAS and genome sequencing technologies. Accordingly, despite the variations related to the *CYP1B1*, various PCG loci have been mapped (over 150 mutations, including missense, nonsense, regulatory, insertions, or deletions). Notably, in PCG, patients usually have geographically related mutations with different severity in the stage of disease and occurrence in a variety of cells [27]. Therefore, the scientific investigation should be conducted in areas not covered entirely in the scientific literature and obtain beneficial information regarding rare disorders, especially in the ophthalmologic area.

Genetic testing and WES for analyzing variants of candidate genes will provide a molecular diagnosis and help effective genetic counseling in PCG and other secondary findings. Moreover, suppose a pathogenic or likely pathogenic variant responsible for an autosomal recessive disorder has been identified in secondary findings. In that case, the preimplantation genetic diagnosis is necessary to prevent the risk of congenital disabilities. However, that would be a potent approach to recognizing most genetic risk factors for the case that might currently affect her life or in the future.

## Figures and Tables

**Table 1.** Details of multiple computational analyses about two candidate single nucleotide variants

	CYP1B1:NM_000104:exon3:c.G1103A:p.R368H (rs79204362)	NTF4:NM_006179:exon2:c.C263T:p.A88V (rs61732310)
Clinvar	Conflicting interpretation of pathogenicity	Variant of uncertain significance
Intervar	Variant of uncertain significance (PP3-PP5-BS1)	Variant of uncertain significance (BS1-PM1-PP2)
Varsome	Variant of uncertain significance (PP5-PP2-PP3-BP6)	Likely- Benign (BS1-BP6-BP1-BP4)
Provean	Deleterious	Neutral
SNP & GO	Disease	Neutral

**Table 2.** Observed incidental/secondary findings of the case

Gene	Variant	State	Disease	Clinvar	Case possibility
*ABCA4*	NM_000350:exon42:c.G5882A:p.G1961E	het	ABCA4 related disorders (AR):	1. Pathogenic/likely pathogenic	Career
			1. Stargardt disease	2. Pathogenic/likely pathogenic	
			2. Cone-rod dystrophy 3	3. Pathogenic	
			3. Retinal dystrophy		
*POLH*	NM_001291969:exon9:c.A1231G:p.K411E	het	Xeroderma pigmentosum (AR)	Pathogenic/likely pathogenic	Career
	NM_006502:exon11:c.A1603G:p.K535E				
*ACADSB*	NM_001330174:exon3:c.C137T:p.T46I	het	Deficiency of 2-methylbutyryl-CoA dehydrogenase (AR)	Pathogenic	Career
	NM_001609:exon4:c.C443T:p.T148I				
*PAH*	NM_000277:exon6:c.G688A:p.V230I	het	Phenylketonuria (AR)	Pathogenic/likely pathogenic	Career
*MEFV*	NM_000243:exon10:c.T2177C:p.V726A	het	Familial Mediterranean fever (AD)	Pathogenic/likely pathogenic	Disease (There is no related phenotype.)
*ACMG *recommendation genes					
*RET*	NM_020630:exon18:c.C2944T:p.R982C NM_020975:exon18:c.C2944T:p.R982C	het	Hirschprung (AD) susceptibility to risk factor	-	-

AR, autosomal dominant; AD, autosomal recessive.
